# Controlling Contact Configuration of Carboxylic Acid-Based Molecular Junctions Through Side Group

**DOI:** 10.1186/s11671-019-3087-7

**Published:** 2019-07-26

**Authors:** Jun-Ren Huang, Hong Huang, Cai-Ping Tao, Ju-Fang Zheng, Ying Yuan, Ze-Wen Hong, Yong Shao, Zhen-Jiang Niu, Jing-Zhe Chen, Xiao-Shun Zhou

**Affiliations:** 1 Key Laboratory of the Ministry of Education for Advanced Catalysis Materials, Institute of Physical Chemistry, Zhejiang Normal University, Jinhua, 321004 China; 2 Department of Physics, Shanghai University, Shanghai, 200444 China; 3 Zhejiang Tianyan Technology Co., Ltd, Hangzhou, 311215 China

**Keywords:** Carboxylic acid, Cu, Scanning tunneling microscopy break junction, Contact configuration, Side-substituted

## Abstract

In this paper, the contact configuration of single molecular junction is controlled through side group, which is explored by electrochemical jump-to-contact STM break junction. The conductance values of 2-methoxy-1,3-benzenedicarboxylic acid (2-M-1,3-BDC) is around 10^–3.65^ G_0_, which is different from that of 5-methoxy-1,3-benzenedicarboxylic acid (5-M-1,3-BDC) with 10^–3.20^ G_0_. Interestingly, the conductance value of 2-M-1,3-BDC is the same as that of 1,3-benzenedicarboxaldehyde (1,3-BDCA), while single molecular junctions of 5-M-1,3-BDC and 1,3-benzenedicarboxylic acid (1,3-BDC) give out similar conductance value. Since 1,3-BDCA binds to the Cu electrode through one oxygen atom, the dominated contact configuration for 1,3-BDC is through two oxygen atoms. The different conductance values between 2-M-1,3-BDC and 5-M-1,3-BDC can be attributed to the different contact configurations caused by the position of the side group. The current work provides a feasible way to control the contact configuration between the anchoring group and the electrode, which may be useful in designing future molecular electronics.

## Background

A well understanding of the electron transport through single molecular junctions is a fundamental interest in the development of molecular electronics [1–14]. In recent years, numerous literature have indicated that the single molecular conductance can be influenced by intrinsic molecular structure [10, 15–18], anchoring groups [19], contact configurations [20, 21], electrode materials [22–24], and so on [4, 14, 25, 26]. Among them, contact configurations play an important role in electron transport of single molecular junctions [27–29]. However, there is rather limited report on this issue, because of the difficulty in controlling the contact configuration.

About contact configuration, some experimental works show multiple sets of conductance values for single molecular junctions corresponding to different contact configurations [20, 30]. However, multiple configurations bring complexity and difficulty in the analysis of the single molecular conductance. The ability to control contact configuration between electrodes and anchoring groups is extremely important, for it can exclude the complexity of contact configurations for future molecular electronics. One way to control the contact configurations is mechanical controlling of single-molecule junctions, and conductance values can be switched between low and high values by mechanically switching of the molecule and electrode contact configurations [31]. Such mechanically controlling may still bring different configurations and is difficult to be used in the future molecular electronics. Recently, the adding of side groups are demonstrated to prevent the molecular conductance from switching during mechanical modulation [28], which shows the possibility of controlling contact configuration through side groups. Therefore, the adding of side groups may provide a feasible way to prevent forming several configurations between molecules and electrodes.

**Fig. 1:**
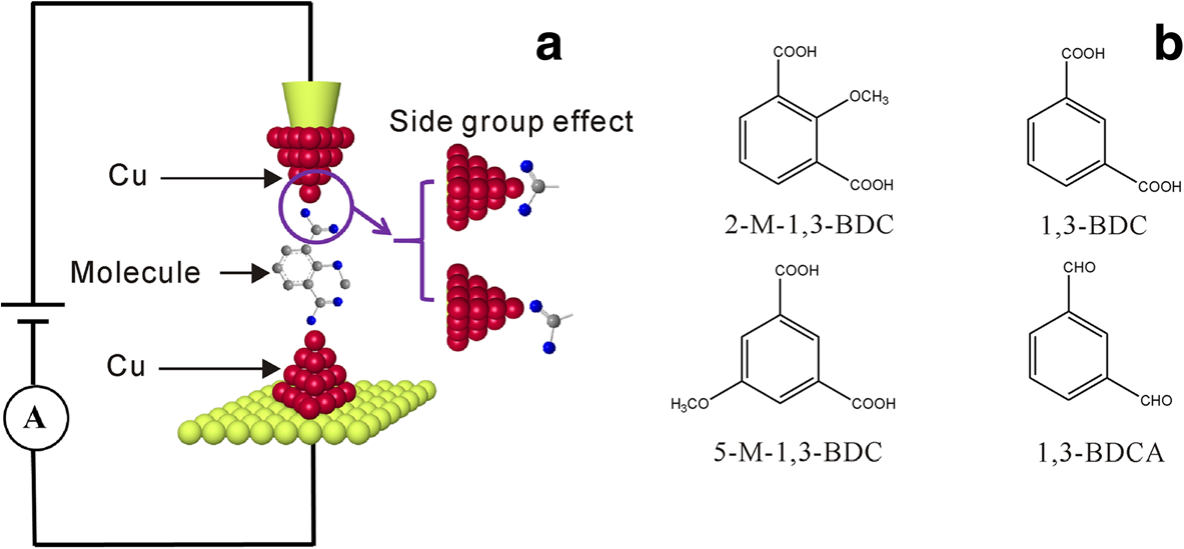
Schematic diagram of electrochemical scanning tunneling microscopy break junction (ECSTM-BJ) and molecular structures. **a** Schematic illustration of the ECSTM-BJ approach for the conductance measurement of single-molecule junctions (red balls, Cu; green balls, Au; blue balls, O; gray balls, C) and **b** the target molecular structure of 2-M-1,3-BDC, 1,3-BDC, 5-M-1,3-BDC, and 1,3-BDCA

Herein, we choose benzene-based carboxylic acid molecules with various side groups as target molecules to investigate the possible contact configurations in single molecular junctions. The carboxylic acid group has been demonstrated to form single molecular junctions with various electrodes [19, 24, 30, 32]. The target molecules include 2-methoxy-1,3-benzenedicarboxylic acid (2-M-1,3-BDC), 1,3-benzenedicarboxylic acid (1,3-BDC), 5-methoxy-1,3-benzenedicarboxylic acid (5-M-1,3-BDC), and 1,3-benzenedicarboxaldehyde (1,3-BDCA) (Fig. 1). Electrochemical jump-to-contact STM break junction (ECSTM-BJ) is used to construct and measure the single molecular junctions with Cu electrodes (Fig. 1). The Cu electrode is chosen, for it can form more effective molecular junctions with carboxylic acid than Au electrode as reported in our previous works [30]. Especially, electrochemical surrounding can prevent the Cu from oxidation, while the single molecular junctions of the carboxylic acid-based molecule cannot be formed with Cu electrode in the air [33].

## Methods

Na_2_SO_4_, CuSO_4_, and 1,3-BDC were purchased from Alfa-Aesar, 2-M-1,3-BDC and 5-M-1,3-BDC were purchased from Sigma-Aldrich, and 1,3-BDCA was obtained from TCI (Tokyo Chemical Industry Co., Ltd.). All of them were used as received. Naturally formed Au (111) on single crystal bead was used as a substrate, while the Pt-Ir insulated by thermosetting polyethylene glue was used as a tip. Pt and Cu wires were used as the counter and reference electrode, respectively.

Conductance measurement of single molecular junctions was carried out on a modified Nanoscope IIIa STM (Veeco, Plainview, NY, USA) and in an aqueous solution containing 1 mM CuSO_4_ + 50 mM Na_2_SO_4_ + 1 mM target molecules. The Pt-Ir tip and Au (111) substrate were set at − 5 and 45 mV versus Cu wire, respectively. In this case, the Cu bulk deposition could occur on the tip but not the substrate. After that, the tip was driven toward the substrate to a sufficiently close distance, and then the jump-to-contact process happened. The tip was pulled away from the substrate at a speed of 20 nm/s. During this process, the conductance trace was recorded till the breaking of single molecular junctions, while Cu clusters were simultaneously produced. Thousands of conductance traces were collected to construct the conductance histogram without data selection. More details for the ECSTM-BJ were reported in our previous works [23, 34, 35].

We carried out the theoretical calculation of the single molecular junction. Standard density functional theory (DFT) method is used to relax the junction structure, where there are 3–4 buffer layers attached to both sides and a large vacuum layer (about 15 Å) inserted outside. Nonequilibrium Green’s function (NEGF) method is adopted to calculate the transport properties, i.e., the transmission coefficients of the junctions at equilibrium [36, 37]. In all the above calculations, Perdew-Burke-Ernzerhof (PBE) functional is used for the exchange-correlation core, and for the sake of both accuracy and efficiency, double-zeta polarized (DZP) basis set is used for organic molecule and the outmost layer of copper atoms and single-zeta polarized (SZP) basis set is used for the other copper layers deep into the electrodes. A (4,4) K-sampling is set along the transverse plane. All the calculations are completed with the open-source package SHINE (Shanghai Integrated Numeric Engineering).

## Results and Discussion

### Single Molecular Conductance of 2-M-1,3-BDC with the Methoxy Side Group on 2-Position of Molecule

**Fig. 2:**
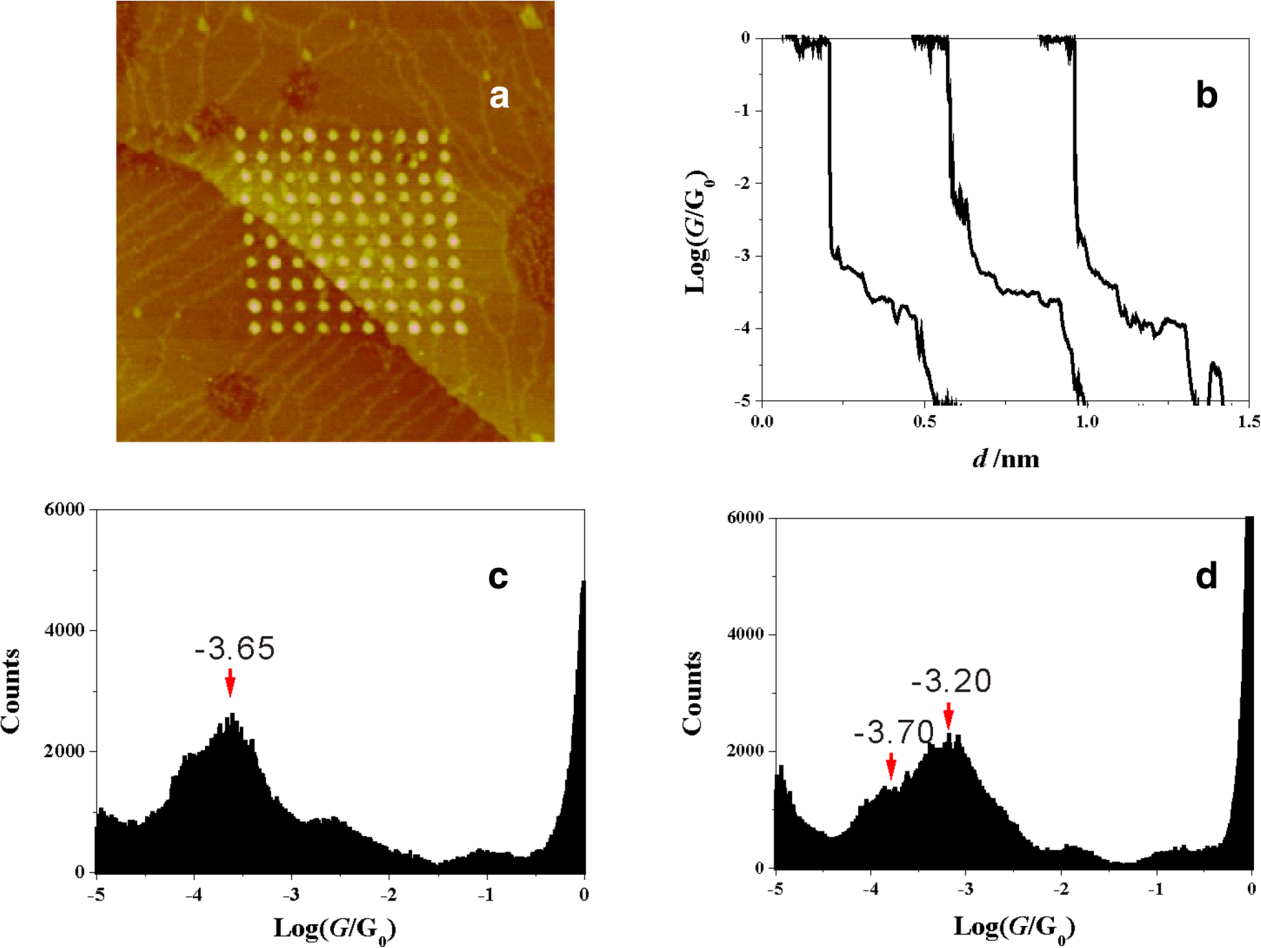
STM image and single molecular conductance for 2-M-1,3-BDC and 1,3-BDC. **a** The STM image (200 × 200 nm^2^) of a 10 × 10 array of Cu clusters forming with the conductance traces simultaneously. **b** Typically conductance traces in solution containing 2-M-1,3-BDC in logarithm scale. Conductance histograms constructed without data selection from 1500 conductance traces measured in solution with **c** 2-M-1,3-BDC and **d** 1,3-BDC

We firstly investigated the single molecular junctions of 2-M-1,3-BDC, which has one methoxy side group on 2-position of 1,3-BDC. The experiment was carried out in an aqueous solution containing 1 mM 2-M-1,3-BDC + 1 mM CuSO_4_ + 50 mM Na_2_SO_4_ by using the ECSTM-BJ approach. Cu clusters were simultaneously produced as a side product (Fig. 2a). Figure 2b displays typical conductance traces in logarithm scale and shows the conductance plateaus of Cu-(2-M-1,3-BDC)-Cu around 10^–3.65^ G_0_. Thousands of conductance traces were collected to construct conductance histogram of 2-M-1,3-BDC without data selection in logarithm scale (Fig. 2c). An obvious peak is found around 10^–3.65^ G_0_, which is consistent with conductance step in conductance traces. Here, the pronounced peak shows the single molecular conductance with the dominated molecule-electrode contact configuration.

Surprisingly, the conductance value of 2-M-1,3-BDC is obviously different from the conductance of 1,3-BDC. Figure 2d displays the conductance histogram of 1,3-BDC and shows dominated conductance peak forming around 10^–3.20^ G_0_, which is similar to a previous report [35]. The methoxy side group cannot bind to the electrode forming effective molecular junctions, thus 2-M-1,3-BDC should bind to the electrode through carboxylic acid anchoring group. The large conductance difference between 2-M-1,3-BDC and 1,3-BDC shows the important role of the methoxy side group on the single-molecule conductance.

The methoxy side group has an effect of pulling electron, which may change the conductance value [38]. However, less than 20% of conductance change is found for molecules with different side groups in the literature (only changing one side group) [38], while the conductance difference is about 300% between 2-M-1,3-BDC and 1,3-BDC. Thus, only pulling an electron effect of the side group cannot cause such a large conductance difference.

### Single Molecular Conductance of 5-M-1,3-BDC with the Methoxy Side Group on the 5-Position of Molecule

In order to further study the important role of the side group, we investigated the single molecular conductance of molecules with methoxy on the 5-position of 1,3-BDC, named 5-M-1,3-BDC. Comparing with 2-M-1,3-BDC, the adding of the side group of methoxy on 5-M-1,3-BDC is away from the anchoring groups.

**Fig. 3:**
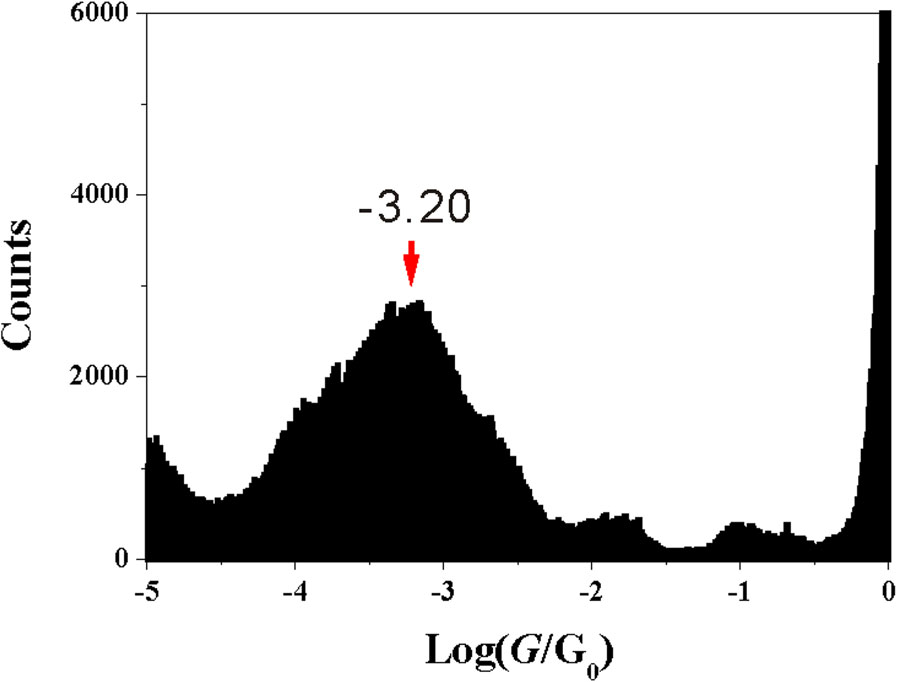
Singe molecular conductance of 5-M-1,3-BDC. The conductance histograms of 5-M-1,3-BDC constructed without data selection from 1500 traces

Figure 3 presents the conductance histograms of 5-M-1,3-BDC, constructing from more than 1000 conductance traces. Comparing with the conductance of 2-M-1,3-BDC, conductance histogram of 5-M-1,3-BDC shows a well-distinguished peak around 10^–3.20^ G_0_ and gives out the same conductance value as that of 1,3-BDC (10^–3.20^ G_0_). This result illustrates that the position of the side group plays a very important role in the single molecular conductance. Though there is the same methoxy group in molecules of 5-M-1,3-BDC and 2-M-1,3-BDC, there are quite different conductance values between them.

### The Possible Reason for Different Conductance Values Among 2-M-1,3-BDC and 5-M-1,3-BDC

What is the reason for a large conductance difference between 2-M-1,3-BDC and 5-M-1,3-BDC? The influence of the side group on destructive quantum interference (DQI) effects in meta-benzene-based molecule might cause this phenomenon [39, 40]. Typically, the conductance of meta-benzene-based molecule is more than one order of magnitude lower than that of a para-benzene-based molecule, while there are other backbones between benzene and anchoring group [41–43]. The substituent effect was theoretically reported on such meta-benzene molecule with DQI, which can largely tune the electron transport of DQI molecules [40]. However, the conductance of meta-benzene-based molecule (1,3-BDC with 10^–3.20^ G_0_) is larger than that of a para-benzene-based molecule (1,4-benzenedicarboxylic acid, 1,4-BDC, with 10^–3.40^ G_0_) [35], showing there is no DQI effect in the 1,3-BDC. DQI is also not found for those molecules having the same backbone but with thiol and amine as anchoring groups [44].

**Fig. 4:**
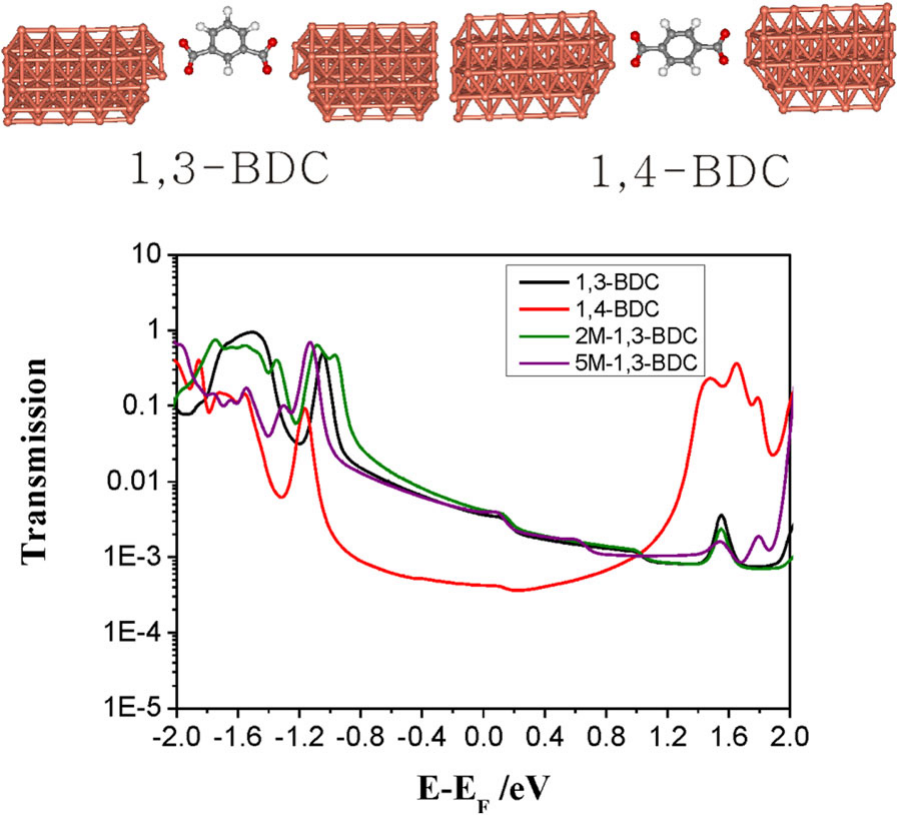
Theoretical calculation of single molecular junctions. Calculated transmission spectra for molecules of 1,3-BDC, 1,4-BDC, 2-M-1,3-BDC, and 5-M-1,3-BDC contacting to Cu electrode through two oxygen atoms of carboxylate

The carboxylic acid can bind to Cu electrode through carbonyl (one oxygen atom) or carboxylate (two oxygen atoms) form, while the dominated peak is contributed to configuration through two oxygen atoms for 1,4-BDC [30]. Our calculations demonstrate that there is no DQI effect in those molecular junctions with contact configurations of anchoring group contacting to Cu electrodes through two oxygen atoms of carboxylate (Fig. 4). No obvious conductance difference is found between 2-M-1,3-BDC and 5-M-1,3-BDC, and the possible reason of DQI influenced by the position of the side group can be ruled out.

Another possibility is that the different dominated contact configuration is formed due to the adding of methoxy on different positions. It was reported that the carboxylic acid can bind to Cu electrode through one oxygen atom or two oxygen atoms form, while the dominated peak is contributed to configuration through two oxygen atoms for 1,4-BDC [30]. Thus the situation may be similar to 1,3-BDC and 5-M-1,3-BDC, and the conductance value of 10^–3.20^ G_0_ might be contributed to the two oxygen atoms (carboxylate) contacting to Cu electrodes. For 2-M-1,3-BDC, the existence of the methoxy side group near the carboxylic acid may prevent single molecular junctions from contacting Cu electrode through two oxygen atoms of carboxylate, and then the conductance value of 10^–3.65^ G_0_ is found. Thus, we may attribute the conductance difference between 2-M-1,3-BDC and 1,3-BDC to the different contact configurations, which is caused by the adding of neighboring methoxy side group. This point is further demonstrated by the conductance measurement of 1,3-BDCA with carbonyl group.

### The Validation of Contact Configuration for 2-M-1,3-BDC by the Measurement of Single Molecular Junctions of 1,3-BDCA

**Fig. 5:**
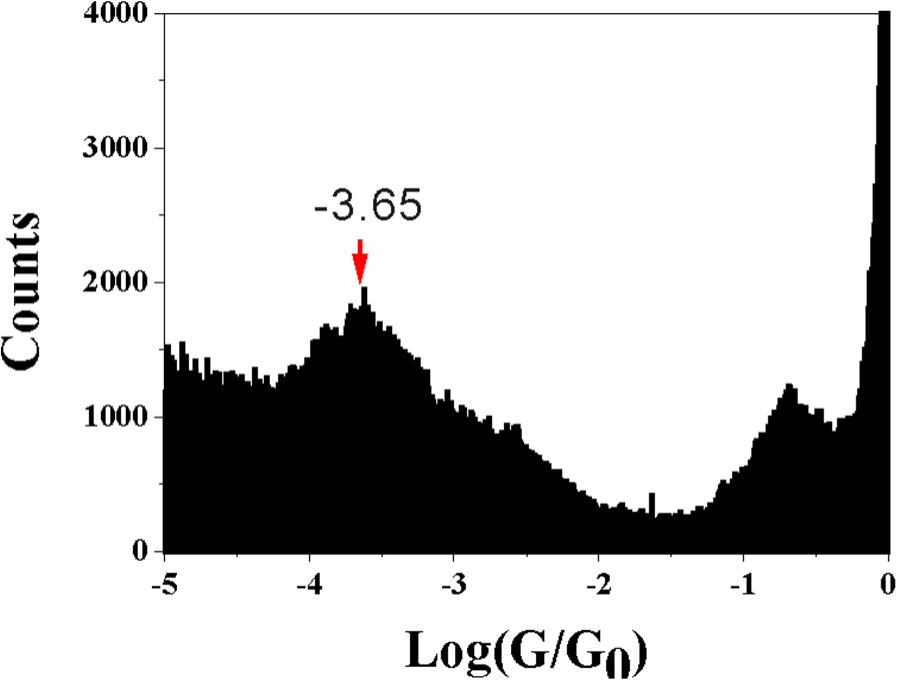
Single molecular conductance of 1,3-BDC. The conductance histogram of 1,3-BDCA constructed from 1100 conductance curves

From above, the neighboring side group has an effect on the single molecular conductance and may influence the contact configuration between carboxylic acid and Cu electrodes. In order to prove this hypothesis, we carried out the conductance measurement of 1,3-BDCA with only carbonyl anchoring group. The carbonyl anchoring group can bind to Cu electrode through one oxygen atom [30, 45]. Figure 5 shows the conductance histogram of 1,3-BDCA with obvious peak located around 10^–3.65^ G_0_. Comparing with the conductance histogram of 1,3-BDC, the conductance of 1,3-BDCA shows a smaller conductance value. However, this value is similar to the conductance of 2-M-1,3-BDC, which may show the same dominated contact configuration formed between 1,3-BDCA and 2-M-1,3-BDC. Especially, we still can find a shoulder peak of 10^–3.70^ G_0_ near the dominated peak value of 10^–3.20^ G_0_ for 1,3-BDC (Fig. 2d). This value (10^–3.70^ G_0_) may be explained by the contact configuration through one oxygen of carboxylate between the anchoring group and the electrode, while dominated peak (10^–3.20^ G_0_) is caused by two oxygens of carboxylate binding to the electrode. Due to the neighboring side group at 2-position, the carboxylate group of 2-M-1,3-BDC fails to form molecular junctions through two oxygens of carboxylate, and only one oxygen from carboxylate group bonds to the electrode.

The conductance values for all studied molecules are summarized in Table 1. The conductance value of 2-M-1,3-BDC is the same as that of 1,3-BDCA, while single molecular junctions of 5-M-1,3-BDC and 1,3-BDC give out a similar conductance value. Since 1,3-BDCA can only bind to the Cu electrode through one oxygen atom, the dominated contact configuration for 1,3-BDC is found through two oxygen atoms. The above conductance values for different molecules show the solid evidence that different contact configurations are formed between 2-M-1,3-BDC and 5-M-1,3-BDC. The adding of methoxy on a neighboring site of anchoring group may have steric hindrance effect, which may forbid the formation of contacting configuration between carboxylic acid and electrode through two oxygen atoms at one or both ends. The current work shows the ability to control the contact configuration through the position of the side group.

**Table 1:** Summary of single molecular conductance for molecules with dominated peak

Molecules	Dominated conductance value (G_0_)
2-M-1,3-BDC	10^–3.65^G_0_
5-M-1,3-BDC	10^–3.20^G_0_
1,3-BDC	10^–3.20^G_0_
1,3-BDCA	10^–3.65^G_0_

## Conclusions

In conclusion, we have measured the single-molecule conductance carboxylic acid-based molecules binding to Cu electrode by using ECSTM-BJ. It is shown that the contact configuration may be controlled by the position of the side group, which can prevent single molecular junctions from contacting Cu electrode through two oxygen atoms of carboxylate for 2-M-1,3-BDC. Such an effect can be invalidated by putting the side group on the 5-position of the molecule (5-M-1,3-BDC). This research provides a feasible way to control the contact configuration between the anchoring group and the electrode, which may be useful in designing future molecular electronics.

## Data Availability

The datasets used and/or analyzed during the current study are available from the corresponding author on reasonable request.

## Abbreviations

1,3-BDC: 1,3-benzenedicarboxylic acid
1,3-BDCA: 1,3-benzenedicarboxaldehyde
1,4-BDC: 1,4-benzenedicarboxylic acid
2-M-1,3-BDC: 2-methoxy-1,3-benzenedicarboxylic acid
5-M-1,3-BDC: 5-methoxy-1,3-benzenedicarboxylic acid
DQI: Destructive quantum interference
ECSTM-BJ: Electrochemical jump-to-contact STM break junction
